# Ramie Elementary Fiber-Reinforced Chitosan Composite Films: Mechanical Properties and Structural Characterization

**DOI:** 10.3390/polym18192413

**Published:** 2026-10-02

**Authors:** Hongbo Li, Jiamin Wang, Pengchen Lu, Yaping Liu, Yu Xian, Yanqing Zhang

**Affiliations:** 1 College of Agricultural Engineering, Shanxi Agricultural University, Taigu 030801, China; 15296695266@163.com (J.W.); 15751452568@163.com (P.L.); yqzhang@sxau.edu.cn (Y.Z.); 2 Shanxi Key Laboratory of Key Technologies and Equipment for Agricultural Machinery, Taigu 030801, China; 3 College of Food Science and Engineering, Shanxi Agricultural University, Taigu 030801, China; liuyaping@sxau.edu.cn; 4 College of Forestry, Shanxi Agricultural University, Taigu 030801, China; xianyu@sxau.edu.cn

**Keywords:** ramie elementary fiber, chitosan composite film, mechanical properties, structural characterization, biodegradable material

## Abstract

Chitosan films have attracted increasing attention as biodegradable packaging materials; however, their relatively low stiffness and limited mechanical stability restrict their practical application. In this study, ramie elementary fiber-reinforced chitosan composite films were prepared using ramie elementary fiber suspensions at concentrations of 0, 0.5, 1.0, and 1.5 g/L. The mechanical properties, chemical interactions, crystalline structure, and surface morphology of the composite films were systematically investigated by tensile testing, Fourier transform infrared spectroscopy, X-ray diffraction, and scanning electron microscopy. The results showed that the incorporation of ramie elementary fibers significantly affected the mechanical behavior of chitosan films. Among the tested concentrations, the composite film with 0.5 g/L ramie elementary fibers exhibited the best overall performance, with the elastic modulus increasing by 130% from 67.46 MPa to 155.14 MPa compared with the pure chitosan film. FTIR analysis indicated that the formation of hydrogen-bonding between ramie elementary fibers and the chitosan matrix, while XRD results showed that the incorporation of ramie elementary fibers enhanced the structural ordering of the chitosan-based composite films without forming a new crystalline phase. SEM observations further confirmed that an appropriate fiber content promoted uniform dispersion and good interfacial bonding, whereas excessive fiber addition led to aggregation, surface roughening, and interfacial defects. These findings demonstrate that low-content ramie elementary fibers can effectively improve the stiffness and structural stability of chitosan composite films, providing a potential strategy for developing biodegradable fiber-reinforced packaging materials.

## 1. Introduction

With the widespread use of conventional petroleum-based plastic packaging materials in daily life, increasing attention has been paid to the environmental pollution caused by their poor degradability. Driven by the growing demand for green and sustainable development, the development of abundant, biodegradable, bio-based, and environmentally friendly composite materials has become an important research direction in the fields of packaging materials and polymer composites [1,2].

Chitosan is derived from chitin and is one of the most abundant natural biopolymers after cellulose [3]. As a biomaterial, chitosan offers several advantages, including wide availability, renewability, good film-forming ability, biocompatibility, and certain antibacterial activity. Therefore, it has great application potential in edible packaging films, fruit and vegetable preservation films, medical dressings, and functional coatings [4]. However, chitosan also has some drawbacks that limit its application, including poor mechanical strength, weak thermal stability, difficulty in forming multilayer structures, and insufficient water resistance [5]. To improve the structural stability and mechanical properties of chitosan films, various modification strategies have been employed, including plasticization, crosslinking, blending, and the incorporation of nanoparticles, natural polysaccharides, cellulose, or plant fibers. These strategies aim to strengthen intermolecular interactions, enhance structural compactness, and improve interfacial compatibility [6,7,8]. Among these strategies, natural plant fibers have attracted increasing attention with the special characters such as low density, good mechanical properties, wide availability, biodegradability, and environmental friendliness [9,10,11,12]. Therefore, they are regarded as important reinforcing phases for developing green chitosan-based composite films and sustainable packaging materials [3,11,13]. Among natural plant fibers, ramie elementary fiber is regarded as a representative high-performance plant fiber due to its high cellulose content, which can reach approximately 68.6–76.2% [14]. Compared with other plant fibers, such as bamboo fiber and flax fiber, ramie elementary fiber generally exhibits superior mechanical properties and toughness, giving it considerable potential for use in natural fiber-reinforced composites [15,16,17]. Moreover, the abundant hydroxy groups on ramie elementary fibers can form hydrogen bonds with the hydroxy and amino groups of chitosan molecular chains, which is beneficial for improving interfacial adhesion between the two phases and facilitating stress transfer within the composite film [5,12]. Recent studies have demonstrated the potential of ramie fiber for reinforcing chitosan-based films. Ji et al. [18] reported that 20 wt% micro ramie fiber slurry increased the tensile strength of chitosan films from 44.6 MPa to 57.8 MPa, while Jadhao et al. [19] achieved a tensile strength of 9.8 MPa at 1% alkali-treated ramie fiber loading. However, both studies used fiber bundles or mechanically fibrillated fibers at relatively high contents, and the reinforcing mechanism at low fiber concentrations—particularly the relationship between hydrogen bonding, crystalline ordering, and the initial elastic modulus—remains insufficiently understood. Moreover, the use of ramie elementary fibers for stiffness regulation of chitosan films has not been systematically investigated.

In this study, ramie bast elementary fibers were extracted by chemical disintegration, and their mechanical properties were tested with the tensile testing method [12]. Chitosan-based composite films reinforced with different contents of ramie elementary fibers were prepared using a casting method. The effects of ramie elementary fiber content on the mechanical properties and reinforcement mechanism of the composite films were systematically investigated via tensile testing, Fourier transform infrared spectroscopy, X-ray diffraction, and scanning electron microscopy. The results provide experimental evidence for evaluating the effect of ramie elementary fiber incorporation on chitosan films and offer a reference for improving the mechanical performance of bio-based packaging materials.

## 2. Materials and Methods

### 2.1. Experimental Materials

Chitosan (degree of deacetylation ≥95%, viscosity: 100–200 mPa·s) was purchased from Macklin Biochemical Technology Co., Ltd. (Shanghai, China). Ramie raw material was collected from Dazhou, Sichuan, China. Glacial acetic acid was purchased from Damao Chemical Reagent Factory (Tianjin, China). Anhydrous ethanol and glycerol were obtained from Tianli Chemical Reagent Co., Ltd. (Tianjin, China) and Fengchuan Chemical Reagent Technology Co., Ltd. (Tianjin, China), respectively. A 30% (*w*/*w*) hydrogen peroxide solution (analytical grade, Yongda Chemical Reagent Co., Ltd., Tianjin, China) and an AB-type epoxy resin adhesive (HY-914, Tianjin Yanhai Chemical Co., Ltd., Tianjin, China) were also used in this study.

### 2.2. Experimental Instruments and Equipment

A continuously variable-magnification stereomicroscope (SZ680B2L, Chongqing Aote Optical Instrument Co., Ltd., Chongqing, China) was used to observe ramie elementary fibers and prepare tensile specimens. A low-speed centrifuge (SC-3610, Anhui Zhongke Zhongjia Scientific Instruments Co., Ltd., Hefei, China) was used to remove air bubbles from the chitosan solution to improve its homogeneity. A high-precision short-fiber microtensile testing system (JSF08, Shanghai Zhongchen Digital Technology Equipment Co., Ltd., Shanghai, China) was used to conduct microtensile tests on ramie elementary fibers. A universal tensile test machine (INSTRON 5544, Instron Corporation, Norwood, MA, USA) was used to perform mechanical tests on the chitosan composite films. A Fourier transform infrared spectrometer (FTIR, 1500, JOSVoK Technology Development Co., Ltd., Tianjin, China) was used to characterize the chemical structure of the chitosan composite films. An X-ray diffractometer (XRD, MiniFlex 600, Rigaku, Japan) was used to analyze the crystalline structure of the chitosan composite films. A scanning electron microscope (SEM, JSM-7500F, JEOL Ltd., Akishima-shi, Tokyo, Japan) was used to observe the surface morphology of the ramie elementary fiber-reinforced chitosan composite films.

### 2.3. Testing the Mechanical Properties of Ramie Elementary Fibers

Ramie elementary fibers were extracted from ramie phloem tissue by chemical desintegration. First, a certain amount of ramie bast tissue was immersed in a maceration solution prepared by mixing 30% hydrogen peroxide and glacial acetic acid at a volume ratio of 1:1. The samples were then treated in a forced-air drying oven at 60 °C for 24 h until the fibers turned white. After maceration, the fibers were removed from the test tubes and rinsed with distilled water until neutral. Finally, the cleaned fibers were dehydrated and preserved in 75% ethanol.

**Figure 1:**
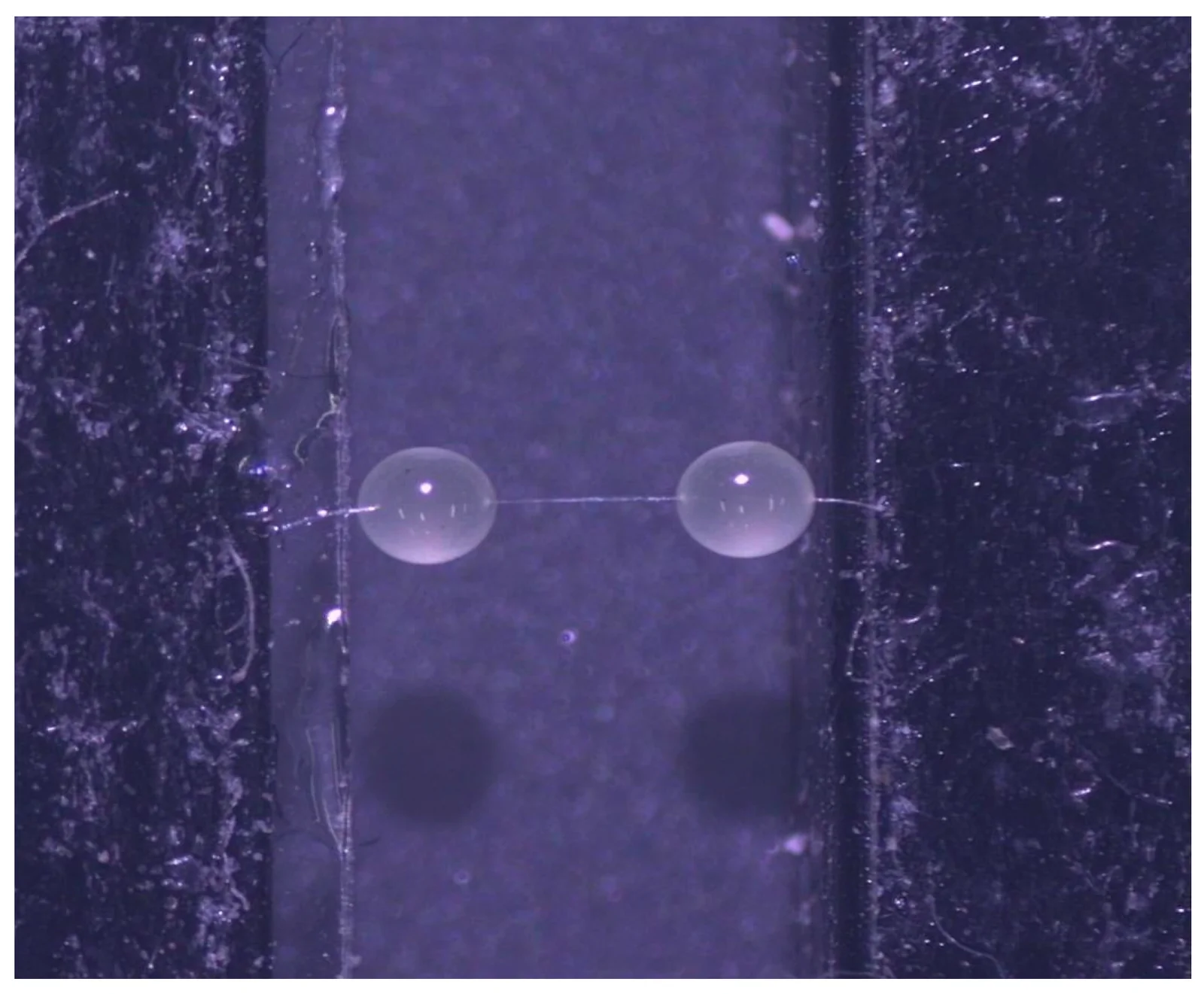
Ramie elementary fiber with epoxy droplets at both ends.

Ramie elementary fibers specimens were prepared as follows. The macerated fiber samples were placed on a glass slide and observed under a stereomicroscope. Straight ramie elementary fibers were carefully selected using fine tweezers and fixed across the groove of an organic glass panel, with both ends attached to adhesive tape. Then, spherical epoxy droplets were fixed at both fiber ends under a stereomicroscope: epoxy AB adhesive was mixed at a ratio of 5:1, and a small amount of adhesive was picked up with the tip of fine tweezers. The adhesive filament was then carefully wound to form two spherical droplets with a diameter of approximately 0.5 mm at both ends of the fiber, with an approximate spacing of 0.7–0.8 mm (Figure 1). Finally, the prepared fiber specimens were cured in a forced-air drying oven at 60 °C for 24 h to fully cure the epoxy droplets. After curing, all fiber specimens were conditioned and stored at 25 °C and 50% RH prior to mechanical tests to minimize the influence of ambient humidity on the fiber properties.

**Figure 2:**
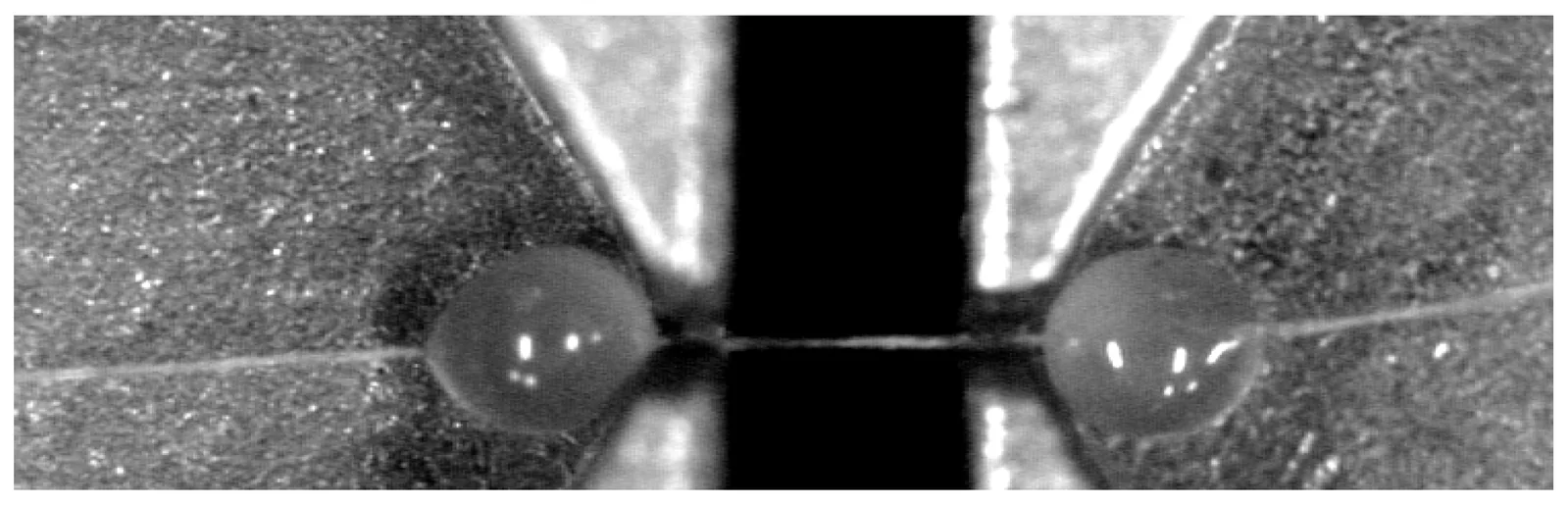
Top view of ramie elementary fiber installed in the fiber clamps.

Tensile tests on ramie elementary fibers was conducted following the guidelines of GB/T Standard Method 35378—201 [20]. After the epoxy droplets had fully cured, the tensile properties of the ramie fibers were measured using a JSF08 high-precision short-fiber microtensile testing system. The cross-section of the ramie fiber was approximately circular, and its diameter and gauge length were determined from top-view images captured by the testing system (Figure 2). For every ramie fiber specimen, its diameter measurements were taken at various locations, and the average value was considered as the diameter of the specimen. The tensile speed was set to 0.0065 mm/s. The stress–strain curves obtained from the tensile tests were used to calculate the elastic modulus, tensile strength, fracture strain, and other mechanical properties of the ramie fibers. At least 15 specimens were tested for the mechanical properties. Fibers with visible damage, kinks, or irregular diameter were excluded.

### 2.4. Preparation and Mechanical Testing of Chitosan Composite Films

After characterizing the mechanical properties of the ramie elementary fibers, chitosan composite films reinforced with these fibers were prepared and evaluated. Chitosan composite films were prepared by the casting method [6]. Chitosan powder was dissolved in 1% acetic acid solution to obtain a 2% chitosan solution, followed by the addition of 1% glycerol as a plasticizer. The mixture was magnetically stirred for 6 h until the chitosan was completely dissolved. The film-forming solution was then transferred into centrifuge tubes and centrifuged at 4000 r/min for 10 min to remove air bubbles and improve film homogeneity and integrity.

The obtained chitosan solution was evenly divided into four groups, with 100 mL in each group. One group was used as the pure chitosan solution, while 0.05, 0.10, and 0.15 g of ramie elementary fibers were added to the other three groups, corresponding to fiber mass concentrations of 0.5, 1.0, and 1.5 g/L, respectively. The mixtures were magnetically stirred for 6 h to ensure uniform dispersion of the fibers in the chitosan matrix. Subsequently, 30 mL of each mixture was poured into a Petri dish with a diameter of 9 cm and dried in an infrared drying oven at 60 °C until the solvent completely evaporated and intact thin films were formed. Finally, the obtained chitosan composite films were stored in a constant-temperature and humidity chamber at 25 °C and 50% RH.

**Figure 3:**
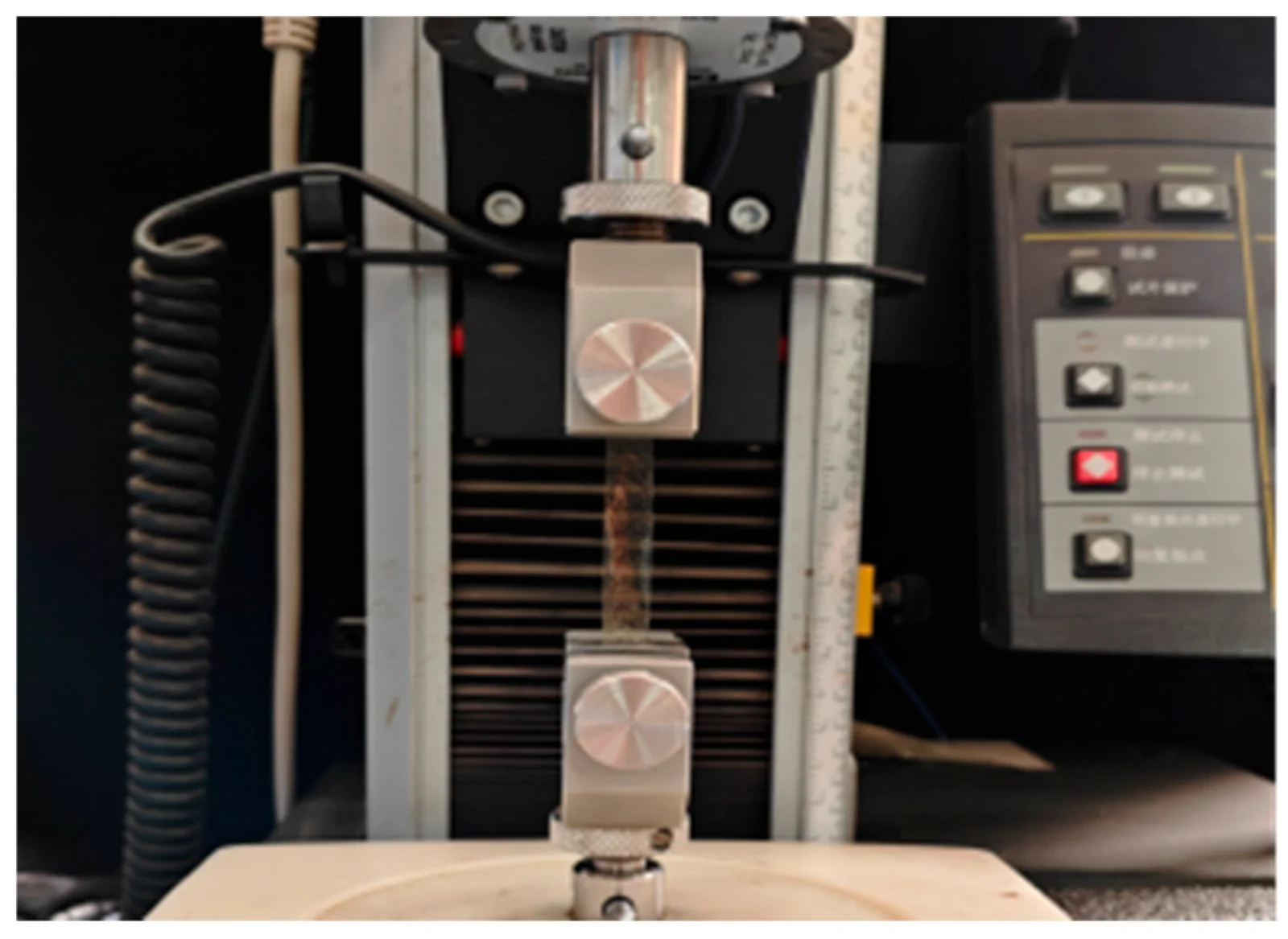
Tensile testing of ramie elementary fiber-reinforced chitosan film.

The mechanical properties of the composite films were tested following the guidelines of ASTM Standard Method D882-18 [21]. The films were first cut into standard tensile specimens with dimensions of 50 mm × 10 mm. Before testing, the thickness of each specimen was measured at five randomly selected points within the gauge section using a high-precision screw micrometer with an accuracy of 0.001 mm, and the average value was used as the specimen thickness. To prevent slipping during the tensile test, both ends of each specimen were wrapped with 400-grit sandpaper before clamping. The longitudinal axis of the specimen was carefully aligned with the tensile direction so as to avoid damage caused by improper clamping (Figure 3). The measured thickness, initial length, and other geometric parameters were then input into the automatic control and data-processing systems of the material testing machine. The tensile speed was set at 2 mm/min. The elastic modulus was calculated from the slope of the initial linear region of the stress–strain curve. Based on the consistent linear behavior observed across all specimens, the strain range of 0–1.0% was used for linear fitting. For each fiber concentration (0, 0.5, 1.0, and 1.5 g/L), 9 rectangular specimens were cut from independently prepared films and tested.

### 2.5. FTIR, XRD, and SEM Structural Characterization of Composite Films

The chemical structure of chitosan composite films reinforced with different amounts of ramie elementary fibers was characterized using FTIR. Before testing, all film samples were dried in an oven to reduce the effect of moisture on the results. Then, 5 mg of pretreated composite film fragments and 150 mg of potassium bromide (KBr) powder were fully ground and mixed in a dry agate mortar. The mixture was then pressed at 10–12 MPa for 1–2 min before testing. The test was performed at a resolution of 2 cm^−1^ over a wavenumber range of 400–4000 cm^−1^.

The crystalline structure of the chitosan composite films reinforced with different amounts of ramie elementary fibers was characterized by XRD. The films were cut into 10 mm × 10 mm specimens and fixed flat in the groove of a standard sample holder to ensure a smooth testing surface. XRD patterns were recorded using a Rigaku MiniFlex 600 (Rigaku Corporation, Akishima-shi, Tokyo, Japan) diffractometer equipped with a D/teX Ultra detector. The measurements were performed in θ–2θ reflection geometry using Cu Kα radiation over a 2θ range of 5–80°, with a step size of 0.02° and a scanning speed of 4°/min. The tube voltage and current were 40 kV and 15 mA, respectively. Fixed slit settings were used, including a divergence slit of 1.25°, a scattering slit of 1.25°, and a receiving slit of 0.3 mm.

The surface morphology of the chitosan composite films reinforced with different amounts of ramie elementary fibers was examined by SEM to analyze the effect of ramie elementary fiber content on the structure of the films. Prior to the observation, film samples were gold-coated and the analysis was conducted with an accelerating voltage of 15 kV.

### 2.6. Data Processing Methods

Statistical analysis was performed by Duncan’s multiple range test and one-way analysis of variance (ANOVA) was conducted to evaluate statistically significant disparities (*p* < 0.05) using SAS 8.0 software.

## 3. Results and Discussion

### 3.1. Mechanical Properties of Ramie Elementary Fibers

**Figure 4:**
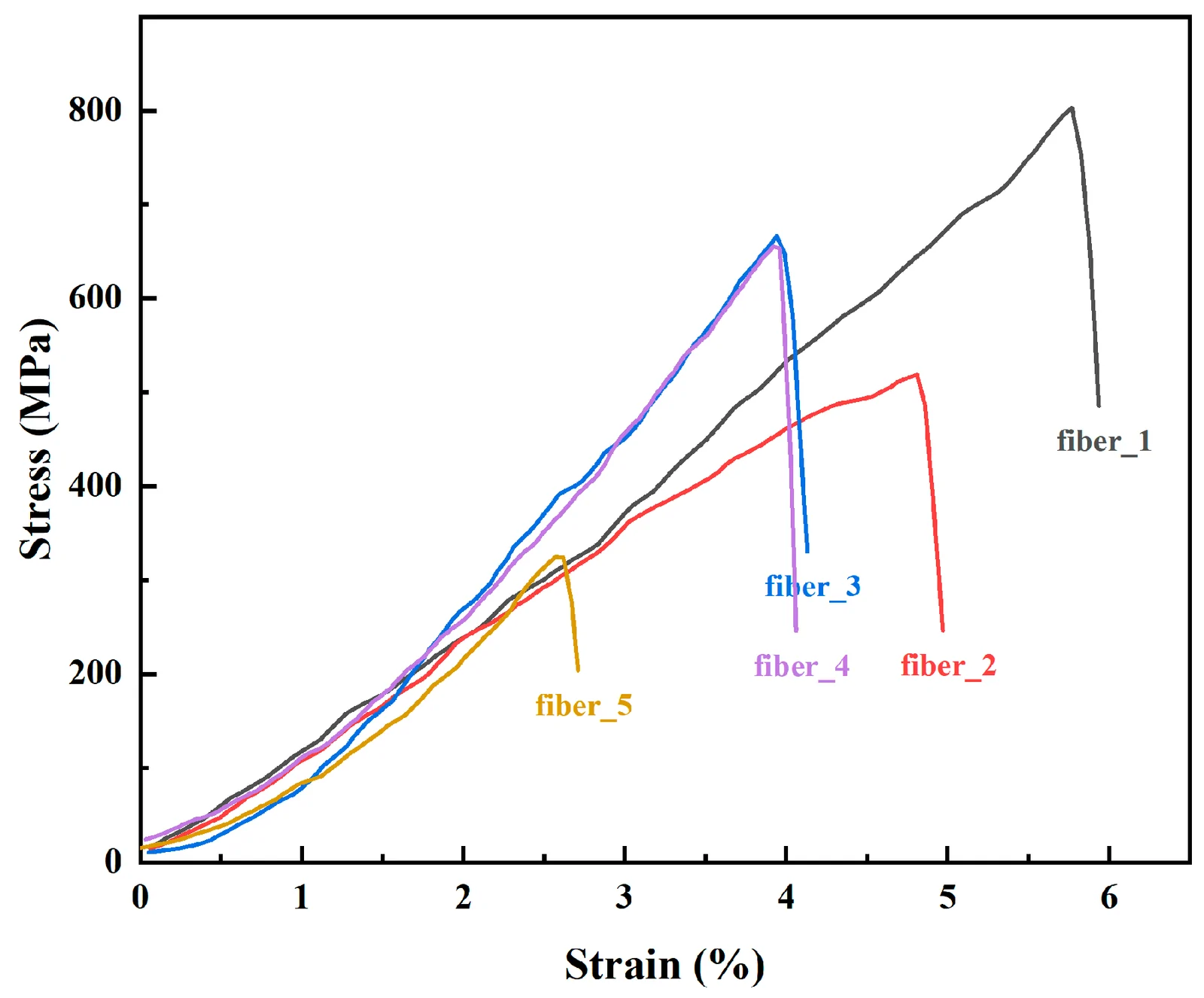
Representative stress–strain curves of ramie elementary fibers.

To evaluate the reproducibility of the tensile tests, at least 15 specimens were tested for each condition. Five representative stress–strain curves are shown in Figure 4, and the corresponding average values and standard deviations are summarized in Table 1. The curve shows that, under tensile loading, the ramie elementary fibers mainly underwent linear elastic deformation followed by brittle fracture, with no obvious yielding or plastic deformation.

With the tensile tests, the mechanical properties of ramie elementary fibers were obtained. The tensile strength, elastic modulus, and fracture strain of ramie elementary fibers were 472.60 ± 183.97 MPa, 14.35 ± 3.89 GPa, and 5.07 ± 0.77%, respectively (*n* = 15). The coefficients of variation were 0.39, 0.27, and 0.15, respectively. These results are generally consistent with those reported by Cao et al. [17], who measured ramie single fibers using a similar microtensile tester and obtained a tensile strength of 1001 MPa, an elastic modulus of 11.4 GPa, and a fracture strain of 8.9%. The tensile strength in the present study (472.60 MPa) is lower than the values (1001 MPa) reported by Cao et al., which is attributed to differences in fiber cross-section area measurement methods and the inherent variability of natural fibers. In this study, the cross-sectional area of ramie fibers was calculated by treating them as having a circular cross-section, whereas in the work of Cao et al., the internal voids of the fibers were removed. The fracture strain measured in this study (5.07%) is lower than the 8.9% reported in these studies, possibly reflecting differences in gauge length and testing conditions.

These findings indicate that ramie elementary fibers possess favorable mechanical properties, suggesting their potential as reinforcing materials for improving the structural stability and mechanical performance of chitosan-based composite films.

### 3.2. Mechanical Properties of Chitosan Composite Films

**Figure 5:**
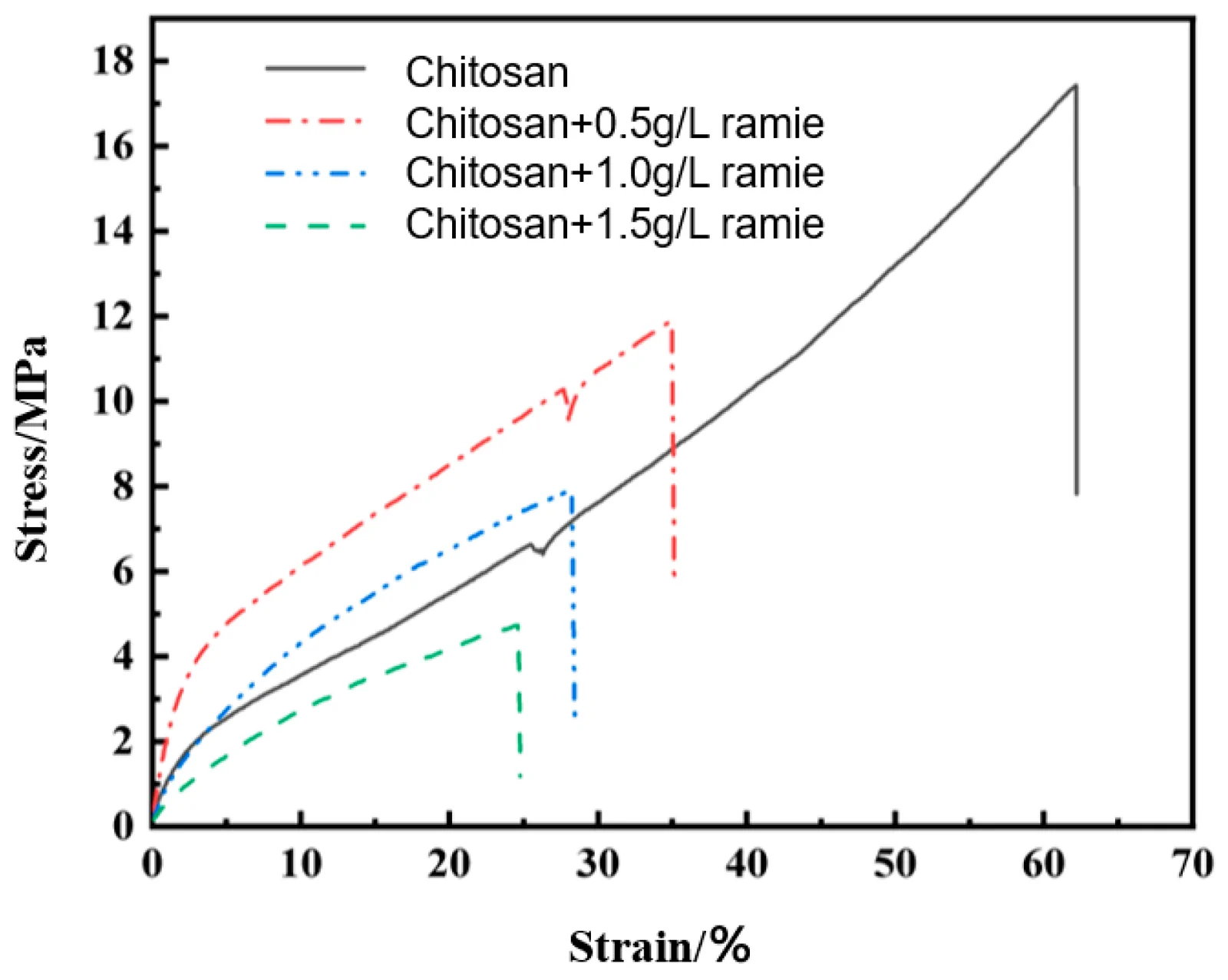
Stress–strain curves of chitosan composite films with different ramie elementary fiber concentrations.

To evaluate the effect of ramie elementary fiber content on the mechanical properties of chitosan films, composite films with different ramie elementary fiber concentrations were prepared and tested according to the procedure described in Section 2.4. The stress–strain curves are shown in Figure 5, and the corresponding mechanical properties are summarized in Table 2.

Pure chitosan films exhibited obvious ductile behavior. As shown in Figure 5, the tensile strength of the pure chitosan films was 16.60 ± 4.26 MPa, and the fracture strain was 63.94 ± 10.78%, indicating good tensile deformability. However, the initial slope of the stress–strain curve was relatively low, with an elastic modulus of only 67.46 ± 14.51 MPa, suggesting limited resistance to elastic deformation.

Compared with pure chitosan films, the composite films containing 0.5 g/L ramie elementary fibers showed a markedly steeper initial slope in the stress–strain curve. The elastic modulus increased to 155.14 ± 44.03 MPa, corresponding to a 130% increase compared with that of the pure chitosan films. Meanwhile, the composite films maintained a fracture strain of 35.47 ± 6.74% and a tensile strength of 12.28 ± 2.53 MPa. These results indicate that although the addition of 0.5 g/L ramie elementary fibers did not improve the tensile strength or fracture strain, it significantly increased the initial elastic modulus. This suggests that a low ramie elementary fiber content can effectively regulate the stiffness of chitosan films.

When the fiber concentration increased to 1.0 g/L, the elastic modulus decreased to 57.23 ± 17.49 MPa, the tensile strength decreased to 6.77 ± 1.76 MPa, and the strain at break decreased to 32.35 ± 4.98%. With a further increase in fiber concentration to 1.5 g/L, the overall mechanical performance of the composite films continued to decline, with the elastic modulus decreasing to 12.56 ± 1.77 MPa, the tensile strength to 2.09 ± 0.29 MPa, and the strain at break to 17.96 ± 2.91%. These results indicate that higher ramie fiber contents not only failed to enhance the mechanical properties of the composite films but also reduced their strength, ductility, and stiffness.

To further validate the statistical significance of the observed differences, one-way ANOVA was performed on the tensile test results (Table 3). Ramie fiber content had a highly significant effect on the elastic modulus (F(3,32) = 38.76, *p* < 0.001), tensile strength (F(3,32) = 80.23, *p* < 0.001), and fracture strain (F(3,32) = 86.23, *p* < 0.001).

Overall, the effect of ramie fibers on the mechanical properties of chitosan films was strongly dependent on fiber content. At low fiber contents (0.5 g/L), ramie fibers may restrict the movement of chitosan molecular chains during small deformation and bear part of the applied load, thereby improving the resistance of the composite films to initial deformation and contributing to their dimensional stability. However, with increasing fiber content, fiber aggregation, interfacial defects, and structural heterogeneity may reduce the load-transfer efficiency between the fibers and the matrix, resulting in decreased strength, ductility, and stiffness. Therefore, the main contribution of ramie fibers is to regulate the initial stiffness of chitosan composite films at low fiber contents, whereas no improvement was observed in tensile strength or strain at break.

Compared with previous studies on ramie fiber-reinforced chitosan films, the mechanical behavior observed in this study exhibits a distinct feature. Ji et al. [18] reported that 20 wt% micro ramie fiber slurry increased the tensile strength of chitosan films from 44.6 MPa to 57.8 MPa, while Jadhao et al. [19] achieved a tensile strength of 9.8 MPa at 1% alkali-treated ramie fiber loading. In both cases, the enhancement was primarily reflected in tensile strength at relatively high fiber contents. In contrast, the present study shows that ramie elementary fibers at a low concentration (0.5 g/L) significantly increased the initial elastic modulus by 130.0% but did not improve the tensile strength. This difference can be attributed to the scale and surface characteristics of the reinforcing phase. The micro ramie fiber slurry used by Ji et al. [18] consisted of fiber bundles or fragments with an average diameter of 3.98 μm, while the alkali-treated ramie fiber used by Jadhao et al. [19] was chopped to approximately 1 mm. Both types of fibers possess relatively low specific surface areas and may act as a macroscopic reinforcing skeleton at high contents. In contrast, ramie elementary fibers are individual fiber cells with a much higher specific surface area and more exposed cellulose hydroxy groups, which facilitate dense hydrogen bonding with chitosan molecular chains at low loading. This dense hydrogen-bonding network restricts the initial movement of chitosan chains under small deformation, thereby enhancing the initial stiffness. However, because the elementary fibers are short and randomly oriented, they cannot effectively bear the applied load after the onset of plastic deformation, which explains why the tensile strength and fracture strain decreased at higher fiber contents. These results indicate that the reinforcing mechanism of ramie elementary fibers in chitosan films is fundamentally different from that of fiber bundles or mechanically fibrillated fibers, and that low-content elementary fibers are more effective for stiffness regulation than for strength enhancement.

### 3.3. Microstructure and Morphology Characterization of Chitosan Composite Films

To further elucidate the reinforcing mechanism suggested by the mechanical results, the chemical interactions, crystalline structure, and surface morphology of the composite films were characterized.

**Figure 6:**
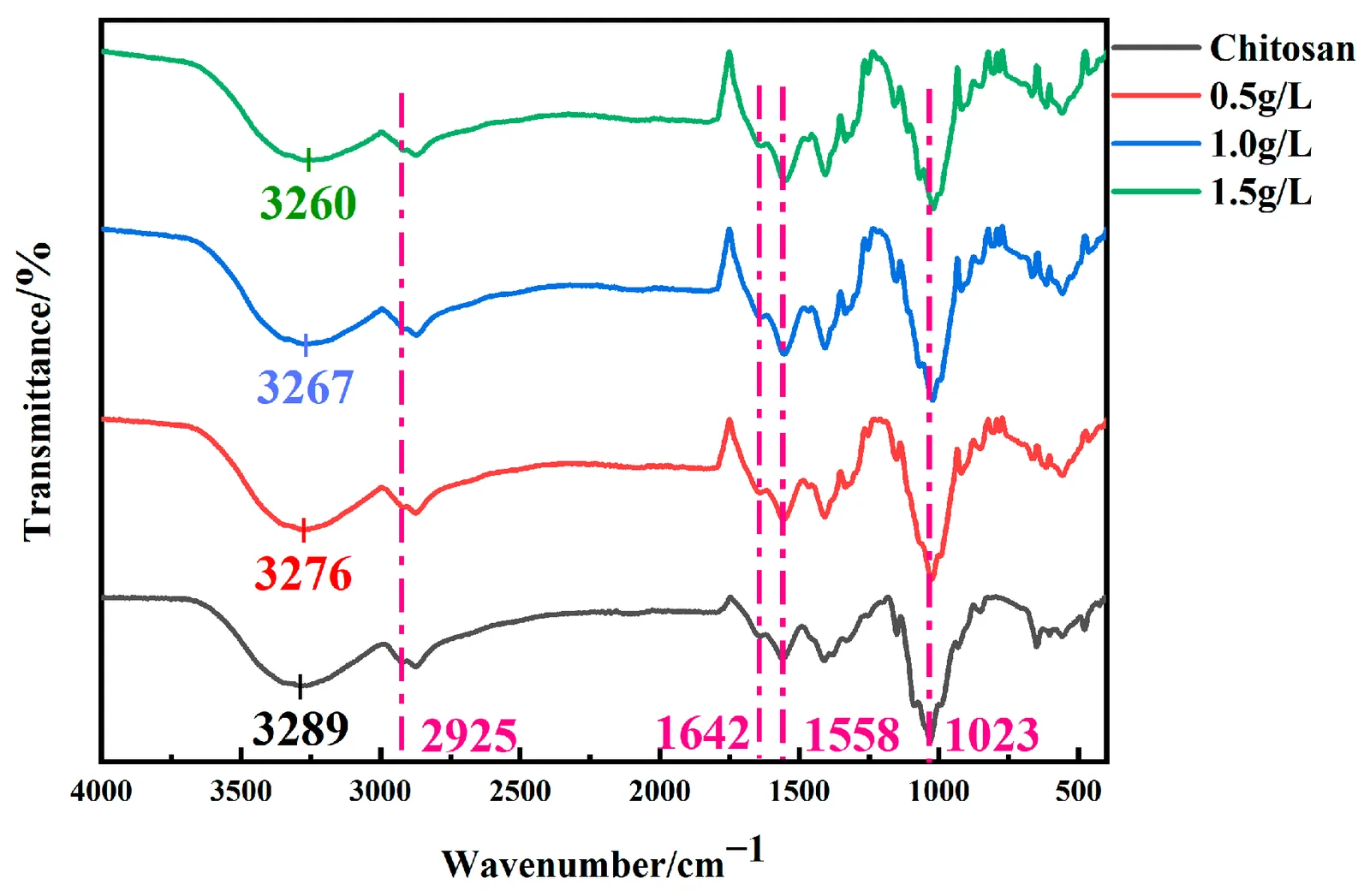
FTIR spectra of pure chitosan film and ramie elementary fiber-reinforced chitosan composite films.

The FTIR results for the pure chitosan film and the composite films reinforced with different ramie elementary fiber concentrations are shown in Figure 6.

The broad absorption band at approximately 3289 cm^−1^ in the pure chitosan film was mainly attributed to the stretching vibrations of O–H and N–H groups. After the incorporation of ramie elementary fibers, this band shifted to lower wavenumbers, indicating the formation of hydrogen bonds between the hydroxy groups of ramie elementary fibers and the hydroxy or amino groups of chitosan molecular chains. In addition, the characteristic absorption bands at around 2925 cm^−1^, 1642 cm^−1^, 1558 cm^−1^, and 1023 cm^−1^ were still observed in the composite films, corresponding to C–H stretching, amide-related vibrations, N–H bending, and C–O/C–O–C stretching vibrations, respectively. No new characteristic bands were detected, suggesting that the interaction between ramie elementary fibers and chitosan was mainly established through intermolecular hydrogen bonding rather than the formation of new covalent bonds.

**Figure 7:**
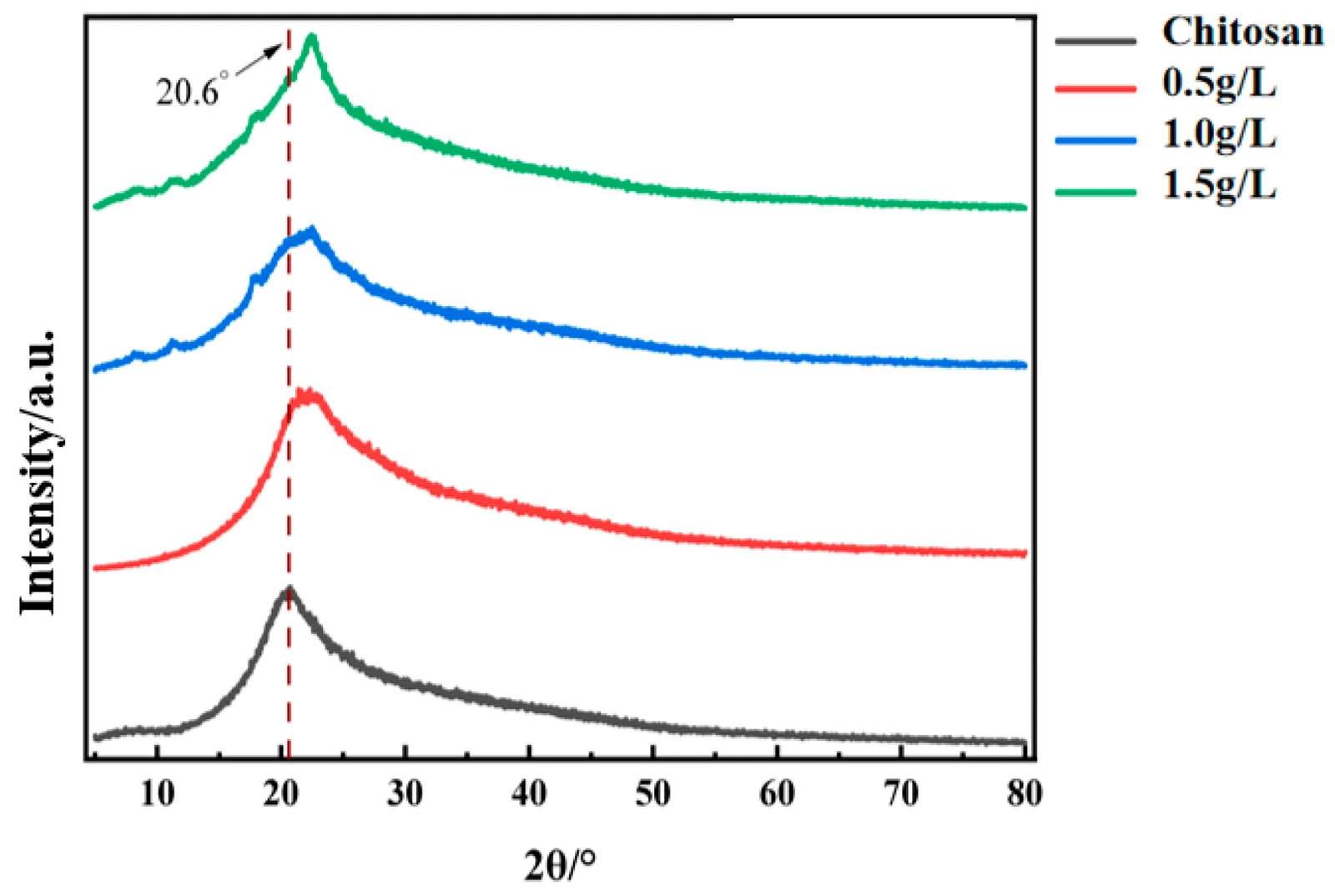
XRD patterns of pure chitosan film and ramie elementary fiber-reinforced chitosan composite films.

Figure 7 presents the XRD patterns of pure chitosan and ramie elementary fiber/chitosan composite films with different ramie elementary fiber concentrations. All samples show a broad diffraction peak at approximately 2θ = 20.6°, which is mainly associated with the semi-crystalline structure of chitosan and may also include overlapping contributions from cellulose in the ramie elementary fibers. Compared with pure chitosan, the composite films exhibit enhanced diffraction intensity around 20.6°. As the ramie elementary fiber concentration increases from 0.5 to 1.5 g/L, this peak becomes more pronounced, indicating that the incorporation of ramie elementary fibers influences the ordered structure of the composite films. This change may be attributed to the presence of crystalline cellulose microfibrils in the ramie elementary fibers and possible intermolecular interactions, such as hydrogen bonding, between cellulose and chitosan chains, which could facilitate a more ordered arrangement of polymer chains in the composite matrix. The characteristic cellulose Iβ peaks at approximately 2θ = 14.6°, 16.9°, and 22.8° [22] are not clearly resolved, likely because of the low fiber content and overlap with the broad chitosan signal. Therefore, the enhanced peak around 20.6° should be interpreted as evidence of improved structural ordering rather than direct evidence of increased cellulose Iβ crystallinity.

**Figure 8:**
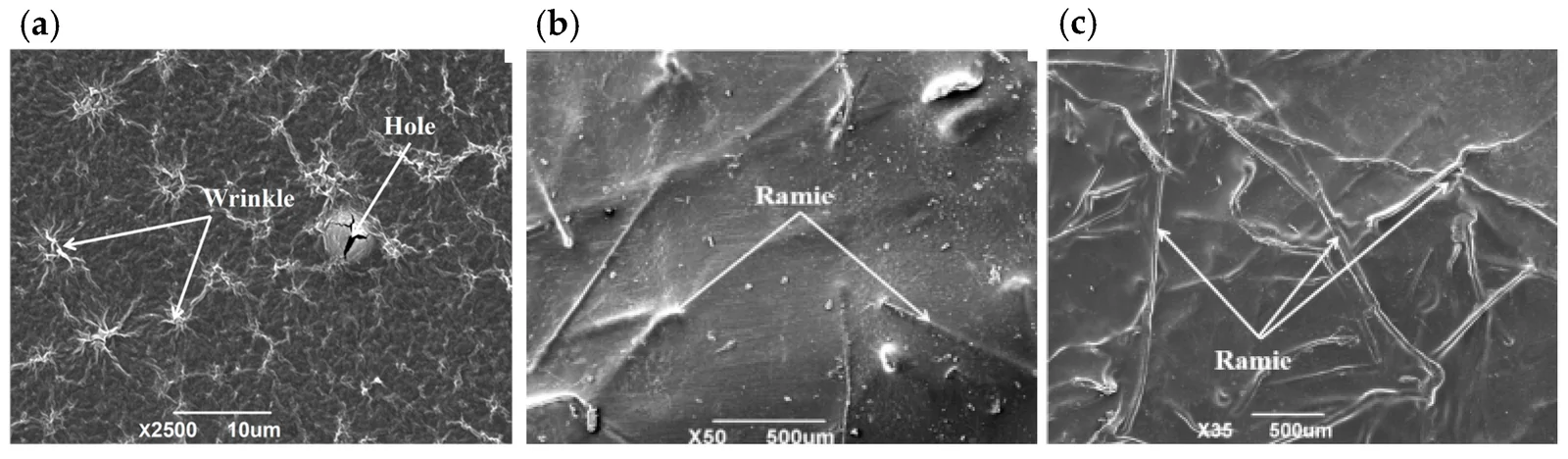
SEM images of ramie elementary fiber/chitosan composite films with different concentrations: (**a**) pure chitosan film; (**b**) composite film with 0.5 g/L ramie elementary fibers and (**c**) composite film with 1.0 g/L ramie elementary fibers.

The surface morphology and fiber dispersion state were then observed by SEM to correlate the microstructural features with the mechanical behavior (Figure 8).

The SEM images showed that the addition of ramie elementary fibers had an obvious modifying effect on the microstructure of chitosan films, but this effect depended strongly on fiber content. The pure chitosan film showed a relatively dense and continuous matrix structure, with local textures, wrinkles, and a few cracks (Figure 8a). This indicates that the pure chitosan film was prone to shrinkage stress during drying and film formation, resulting in brittleness and limited crack resistance.

After the addition of 0.5 g/L ramie elementary fibers, the surface of the composite film became generally smoother, and the fibers or fine particles were relatively well dispersed and embedded in the chitosan matrix (Figure 8b). No obvious large-area aggregation or severe pores were observed, suggesting good compatibility and interfacial bonding between the fibers and chitosan at a low addition level. The fibers acted as a dispersed reinforcing phase in the matrix, helping to transfer stress, hinder crack propagation, and improve the structural stability of the film. Therefore, this fiber concentration was beneficial for improving the mechanical properties and structural integrity of the film.

In contrast, when the ramie elementary fiber content was increased to 1.0 g/L, a large number of long fibrous protrusions, interlaced networks, and obvious wrinkles appeared on the film surface, with some fibers locally exposed or aggregated (Figure 8c). This indicates that excessive fibers were unevenly dispersed in the chitosan matrix, leading to more interfacial defects. Although a higher fiber content may form a more obvious reinforcing skeleton, fiber entanglement and aggregation can disrupt the uniform and continuous structure of the film and form stress concentration points. These defects may reduce the compactness, transparency, and fracture strain of the film and may even affect its barrier properties.

Overall, the addition of 0.5 g/L ramie elementary fibers was more favorable for forming a uniform and dense chitosan composite film with good interfacial bonding, whereas the addition of 1.0 g/L was relatively excessive and tended to cause uneven fiber distribution and surface roughening. Therefore, an appropriate amount of ramie elementary fibers can improve the structure and reinforcing effect of chitosan films, while excessive addition may weaken the uniformity and overall performance of the composite film.

Combined analysis of the stress–strain curves (Figure 5) and SEM morphology (Figure 8) further showed that, under tensile loading, the pure chitosan film exhibited a sudden stress drop at a strain of approximately 26%, indicating the possible occurrence of local yielding, microcrack propagation, or structural damage within the film. After the addition of 0.5 g/L ramie elementary fibers, the composite film still showed a similar stress-drop phenomenon during stretching. However, the corresponding stress value was higher than that of the pure chitosan film, and the strain increased to approximately 28%. This indicates that a small amount of ramie elementary fibers can fill pores in the chitosan matrix, improve structural compactness, and enhance load transfer, thereby increasing the stiffness and load-bearing capacity of the film and, to some extent, delaying local yielding or microcrack propagation. With further increases in fiber concentration, no obvious stress-drop phenomenon was observed in the composite films, suggesting a change in tensile failure behavior. This may be related to the formation of a supporting framework and enhanced interfacial interactions at higher fiber contents, although uneven fiber dispersion or local aggregation may also have contributed. Overall, low contents of ramie elementary fibers can improve the stiffness and load-bearing capacity of the composite films while maintaining the ductile deformation characteristics of the chitosan matrix and delaying the occurrence of local damage. These observations are consistent with the mechanical results, in which the 0.5 g/L composite film exhibited the highest elastic modulus, suggesting that pore filling and improved interfacial bonding at low fiber content were the main contributors to the enhanced stiffness.

## 4. Conclusions

In this study, chitosan composite films reinforced with ramie elementary fibers at concentrations of 0, 0.5, 1.0, and 1.5 g/L were prepared and tested. Tensile results showed that the addition of ramie elementary fibers significantly affected the mechanical properties of the films. The composite film containing 0.5 g/L ramie elementary fibers exhibited the highest elastic modulus among the tested groups, reaching 155.14 MPa, approximately 130% higher than that of the pure chitosan film.

FTIR, XRD, and SEM analyses showed that ramie elementary fibers interacted with the chitosan matrix through hydrogen bonding and promoted partial structural ordering. The reinforcing effect was not a simple superposition of fiber strength and matrix strength, but resulted from the combined effects of fiber properties, fiber scale, interfacial interactions, and dispersion state. An appropriate fiber content, particularly 0.5 g/L, effectively enhanced the stiffness of the composite films by enhancing hydrogen bonding, increasing structural ordering, and filling pores or loose regions in the film.

This study mainly focused on mechanical performance and microstructural characterization, while other functional properties, including water vapor permeability, hydrophobicity, and antibacterial activity, require further investigation.

## Figures and Tables

**Table 1:** Mechanical properties of ramie elementary fibers.

Types	Tensile Strength (MPa)	Elastic Modulus (GPa)	Fracture Strain (%)	Failure Load (mN)
Max	803.34	20.09	6.27	633.34
Min	242.31	7.21	3.95	138.96
Average	472.60	14.35	5.07	288.52
Stdev	183.97	3.89	0.77	147.71
CV	0.39	0.27	0.15	0.51

**Table 2:** Mechanical properties of ramie elementary fiber-reinforced chitosan composite films.

Ramie Fiber Content (g/L)	Elastic Modulus (MPa)	Tensile Strength (MPa)	Fracture Strain (%)
0	67.46 ± 14.51 ^b^	16.60 ± 4.26 ^a^	63.94 ± 10.78 ^a^
0.5	155.14 ± 44.03 ^a^	12.28 ± 2.53 ^b^	35.47 ± 6.74 ^b^
1.0	57.23 ± 17.49 ^b^	6.77 ± 1.76 ^c^	32.35 ± 4.98 ^b^
1.5	12.56 ± 1.77 ^c^	2.09 ± 0.29 ^d^	17.96 ± 2.91 ^c^

Values are expressed as mean ± standard deviation (*n* = 9). Different lowercase letters within the same column indicate significant differences at the 0.05 level according to Duncan’s multiple range test.

**Table 3:** One-way ANOVA of tensile test results for chitosan composite films with different ramie fiber contents.

Source of Variation	df	F-Value	*p*-Values
Elastic modulus	3, 32	38.76	<0.001
Tensile Strength	3, 32	80.23	<0.001
Fracture strain	3, 32	86.23	<0.001

df = degrees of freedom. *p* < 0.01 indicates highly significant, 0.01 ≤ *p* < 0.05 indicates significant, and *p* > 0.05 indicates not significant according to one-way ANOVA.

## Data Availability

Data are contained within the article.
